# Campylobacter jejuni--an emerging foodborne pathogen.

**DOI:** 10.3201/eid0501.990104

**Published:** 1999

**Authors:** S F Altekruse, N J Stern, P I Fields, D L Swerdlow

**Affiliations:** U.S. Food and Drug Administration, Blacksburg, Virginia, USA. saltekru@vt.edu

## Abstract

Campylobacter jejuni is the most commonly reported bacterial cause of foodborne infection in the United States. Adding to the human and economic costs are chronic sequelae associated with C. jejuni infection--Guillian-Barré syndrome and reactive arthritis. In addition, an increasing proportion of human infections caused by C. jejuni are resistant to antimicrobial therapy. Mishandling of raw poultry and consumption of undercooked poultry are the major risk factors for human campylobacteriosis. Efforts to prevent human illness are needed throughout each link in the food chain.


### *Campylobacter jejuni*—An Emerging Foodborne Pathogen

**Sean F. Altekruse,* Norman J. Stern,† Patricia I. Fields,‡ and David L. Swerdlow‡**
*U.S. Food and Drug Administration, Blacksburg, Virginia, USA; †U.S. Department of Agriculture, Athens, Georgia, USA; and ‡Centers for Disease Control and Prevention, Atlanta, Georgia, USA

*Campylobacter jejuni* is the most commonly reported bacterial cause of foodborne infection in the United States. Adding to the human and economic costs are chronic sequelae associated with *C. jejuni* infection—Guillian-Barré syndrome and reactive arthritis. In addition, an increasing proportion of human infections caused by *C. jejuni* are resistant to antimicrobial therapy. Mishandling of raw poultry and consumption of undercooked poultry are the major risk factors for human campylobacteriosis. Efforts to prevent human illness are needed throughout each link in the food chain.

### History

Awareness of the public health implications of *Campylobacter* infections has evolved over more than a century (1). In 1886, Escherich observed organisms resembling campylobacters in stool samples of children with diarrhea. In 1913, McFaydean and Stockman identified campylobacters (called related *Vibrio*) in fetal tissues of aborted sheep (1). In 1957, King described the isolation of related *Vibrio* from blood samples of children with diarrhea, and in 1972, clinical microbiologists in Belgium first isolated campylobacters from stool samples of patients with diarrhea (1). The development of selective growth media in the 1970s permitted more laboratories to test stool specimens for *Campylobacter*. Soon *Campylobacter* spp. were established as common human pathogens. *Campylobacter jejuni* infections are now the leading cause of bacterial gastroenteritis reported in the United States (2). In 1996, 46% of laboratory-confirmed cases of bacterial gastroenteritis reported in the Centers for Disease Control and Prevention/U.S. Department of Agriculture/Food and Drug Administration Collaborating Sites Foodborne Disease Active Surveillance Network were caused by *Campylobacter* species. Campylobacteriosis was followed in prevalence by salmonellosis (28%), shigellosis (17%), and *Escherichia coli* O157 infection (5%) (Figure 1).

Address for correspondence: Sean Altekruse, Virginia-Maryland Regional College of Veterinary Medicine, Duckpond Road, Blacksburg, VA, 24060, USA; fax: 540-231-7367; e-mail: saltekru@vt.edu.

### Disease Prevalence

In the United States, an estimated 2.1 to 2.4 million cases of human campylobacteriosis (illnesses ranging from loose stools to dysentery) occur each year (2). Commonly reported symptoms of patients with laboratory-confirmed infections (a small subset of all cases) include diarrhea, fever, and abdominal cramping. In one study, approximately half of the patients with laboratory-confirmed campylobacteriosis reported a history of bloody diarrhea (3). Less frequently,

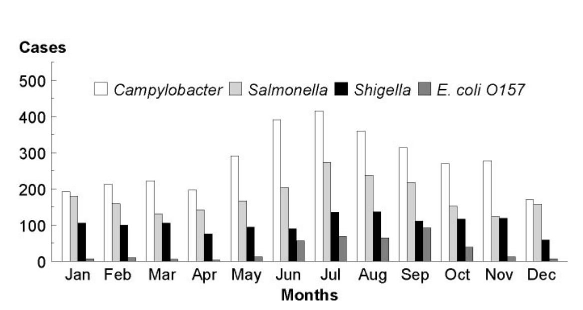

Figure 1. Cases of *Campylobacter* and other foodborne infections by month of specimen collection; Centers for Disease Control and Prevention/U.S. Department of Agriculture/Food and Drug Administration Collaborating Sites Foodborne Disease Active Surveillance Network, 1996.

*C. jejuni* infections produce bacteremia, septic arthritis, and other extraintestinal symptoms (4). The incidence of campylobacteriosis in HIV-infected patients is higher than in the general population. For example, in Los Angeles County between 1983 and 1987, the reported incidence of campylobacteriosis in patients with AIDS was 519 cases per 100,000 population, 39 times higher than the rate in the general population. (5). Common complications of campylobacteriosis in HIV-infected patients are recurrent infection and infection with antimicrobial-resistant strains (6). Deaths from *C. jejuni* infection are rare and occur primarily in infants, the elderly, and patients with underlying illnesses (2).

### Sequelae to Infection

Guillain-Barré syndrome (GBS), a demyelating disorder resulting in acute neuromuscular paralysis, is a serious sequela of *Campylobacter* infection (7). An estimated one case of GBS occurs for every 1,000 cases of campylobacteriosis (7). Up to 40% of patients with the syndrome have evidence of recent *Campylobacter* infection (7). Approximately 20% of patients with GBS are left with some disability, and approximately 5% die despite advances in respiratory care. Campylobacteriosis is also associated with Reiter syndrome, a reactive arthropathy. In approximately 1% of patients with campylobacteriosis, the sterile postinfection process occurs 7 to 10 days after onset of diarrhea (8). Multiple joints can be affected, particularly the knee joint. Pain and incapacitation can last for months or become chronic.

Both GBS and Reiter syndrome are thought to be autoimmune responses stimulated by infection. Many patients with Reiter syndrome carry the HLA B27 antigenic marker (8). The pathogenesis of GBS (9) and Reiter syndrome is not completely understood.

### Treatment of *C. jejuni* Infections

Supportive measures, particularly fluid and electrolyte replacement, are the principal therapies for most patients with campylobacteriosis (10). Severely dehydrated patients should receive rapid volume expansion with intravenous fluids. For most other patients, oral rehydration is indicated. Although *Campylobacter* infections are usually self limiting, antibiotic therapy may be prudent for patients who have high fever, bloody diarrhea, or more than eight stools in 24 hours; immunosuppressed patients, patients with bloodstream infections, and those whose symptoms worsen or persist for more than 1 week from the time of diagnosis. When indicated, antimicrobial therapy soon after the onset of symptoms can reduce the median duration of illness from approximately 10 days to 5 days. When treatment is delayed (e.g., until *C. jejuni* infection is confirmed by a medical laboratory), therapy may not be successful (10). Ease of administration, lack of serious toxicity, and high degree of efficacy make erythromycin the drug of choice for *C. jejuni* infection; however, other antimicrobial agents, particularly the quinolones and newer macrolides including azithromycin, are also used.

### Antimicrobial Resistance

The increasing rate of human infections caused by antimicrobial-resistant strains of *C. jejuni* makes clinical management of cases of campylobacteriosis more difficult (11,12). Antimicrobial resistance can prolong illness and compromise treatment of patients with bacteremia. The rate of antimicrobial-resistant enteric infections is highest in the developing world, where the use of antimicrobial drugs in humans and animals is relatively unrestricted. A 1994 study found that most clinical isolates of *C. jejuni* from U.S. troops in Thailand were resistant to ciprofloxacin. Additionally, nearly one third of isolates from U.S. troops located in Hat Yai were resistant to azithromycin (11). In the industrialized world, the emergence of fluoroquinolone-resistant strains of *C. jejuni* illustrates the need for prudent antimicrobial use in food-animal production (12). Experimental evidence demonstrates that fluoroquinolone-susceptible *C. jejuni* readily become drug-resistant in chickens when these drugs are administered (13). After flouroquinolone use in poultry was approved in Europe, resistant *C. jejuni* strains emerged rapidly in humans during the early 1990s (12). Similarly, within 2 years of the 1995 approval of fluoroquinolone use for poultry in the United States, the number of domestically acquired human cases of ciprofloxacin-resistant campylobacteriosis doubled in Minnesota (14). In a 1997 study conducted in Minnesota, 12 (20%) of 60 *C. jejuni* isolates obtained from chicken purchased in grocery stores were ciprofloxacin-resistant (14).

### Pathogenesis

The pathogenesis of *C. jejuni* infection involves both host- and pathogen-specific factors. The health and age of the host (2) and *C. jejuni*-specific humoral immunity from previous exposure (15) influence clinical outcome after infection. In a volunteer study, *C. jejuni* infection occurred after ingestion of as few as 800 organisms (16). Rates of infection increased with the ingested dose. Rates of illness appeared to increase when inocula were ingested in a suspension buffered to reduce gastric acidity (16).

Many pathogen-specific virulence determinants may contribute to the pathogenesis of *C. jejuni* infection, but none has a proven role (17). Suspected determinants of pathogenicity include chemotaxis, motility, and flagella, which are required for attachment and colonization of the gut epithelium (Figure 2) (17). Once colonization occurs, other possible virulence determinants are iron acquisition, host cell invasion, toxin production, inflammation and active secretion, and epithelial disruption with leakage of serosal fluid (17).

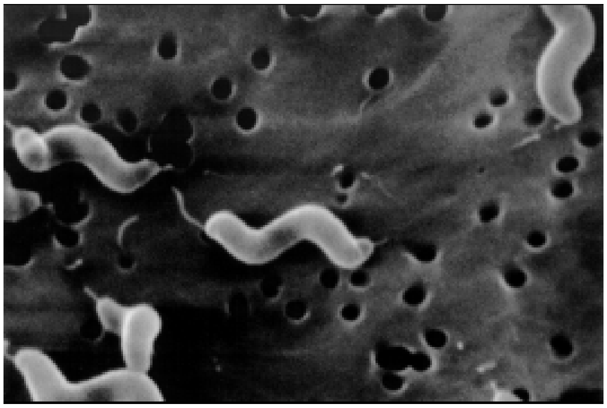

Figure 2. Scanning electron microscope image of *Campylobacter jejuni*, illustrating its corkscrew appearance and bipolar flagella. Source: Virginia-Maryland Regional College of Veterinary Medicine, Blacksburg, Virginia.

### Survival in the Environment

Survival of *C. jejuni* outside the gut is poor, and replication does not occur readily (17). *C. jejuni* grows best at 37°C to 42°C (18), the approximate body temperature of the chicken (41°C to 42°C). *C. jejuni* grows best in a low oxygen or microaerophilic environment, such as an atmosphere of 5% $O_2$, 10% $CO_2$, and 85% $N_2$. The organism is sensitive to freezing, drying, acidic conditions (pH $\leq$ 5.0), and salinity.

### Sample Collection and Transport

If possible, stool specimens should be chilled (not frozen) and submitted to a laboratory within 24 hours of collection. Storing specimens in deep, airtight containers minimizes exposure to oxygen and desiccation. If a specimen cannot be processed within 24 hours or is likely to contain small numbers of organisms, a rectal swab placed in a specimen transport medium (e.g., Cary-Blair) should be used. Individual laboratories can provide guidance on specimen handling procedures (18).

Numerous procedures are available for recovering *C. jejuni* from clinical specimens (18). Direct plating is cost-effective for testing large numbers of specimens; however, testing sensitivity may be reduced. Preenrichment (raising the temperature from 36°C to 42°C over several hours), filtration, or both are used in some laboratories to improve recovery of stressed *C. jejuni* organisms from specimens (e.g., stored foods or swabs exposed to oxygen) (19). Isolation can be facilitated by using selective media containing antimicrobial agents, oxygen quenching agents, or a low oxygen atmosphere, thus decreasing the number of colonies that must be screened (18,19).

### Subtyping of Isolates

No standard subtyping technique has been established for *C. jejuni*. Soon after the organism was described, two serologic methods were developed, the heat-stable or somatic O antigen (20) and the heat-labile antigen schemes (21). These typing schemes are labor intensive, and their use is limited almost exclusively to reference laboratories. Many different DNA-based subtyping schemes have been developed, including pulsed-field gel electrophoresis (PFGE) and randomly amplified polymorphic DNA (RAPD) analysis (22). Various typing schemes have been developed on the basis of the sequence of *fla*A, encoding flagellin (23); however, recent evidence suggests that this locus may not be representative of the entire genome (24).

### Transmission to Humans

Most cases of human campylobacteriosis are sporadic. Outbreaks have different epidemiologic characteristics from sporadic infections (2). Many outbreaks occur during the spring and autumn (2). Consumption of raw milk was implicated as the source of infection in 30 of the 80 outbreaks of human campylobacteriosis reported to CDC between 1973 and 1992. Outbreaks caused by drinking raw milk often involve farm visits (e.g., school field trips) during the temperate seasons. In contrast, sporadic *Campylobacter* isolates peak during the summer months (Figure 1). A series of case-control studies identified some risk factors for sporadic campylobacteriosis, particularly handling raw poultry (25,26) and eating undercooked poultry (27-31) (Table). Other risk factors accounting for a smaller proportion of sporadic illnesses include drinking untreated water (29); traveling abroad (25); eating barbequed pork (28) or sausage (27); drinking raw milk (29, 32) or milk from bird-pecked bottles (33); and contact with dogs (27) and cats (29,31), particularly juvenile pets or pets with diarrhea (25,34). Person-to-person transmission is uncommon (25,32). Overlap is reported between serotypes of *C. jejuni* found in humans, poultry, and cattle, indicating that foods of animal origin may play a major role in transmitting *C. jejuni* to humans (35).

In the United States, infants have the highest age-specific *Campylobacter* isolation rate, approximately 14 per 100,000 person years. As children get older, isolation rates decline to approximately 4 per 100,000 person years for young adolescents. A notable feature of the epidemiology of human campylobacteriosis is the high isolation rate among young adults, approximately 8 per 100,000 person years. Among middle-aged and older adults, the isolation rate is < 3 per 100,000 person years (2). The peak isolation rate in neonates and infants is attributed in part to susceptibility on first exposure and to the low threshold for seeking medical care for infants (2). The high rate of infection during early adulthood, which is pronounced among men, is thought to reflect poor food-handling practices in a population that, until recently, relied on others to prepare meals (2).

### Reservoirs

The ecology of *C. jejuni* involves wildlife reservoirs, particularly wild birds. Species that carry *C. jejuni* include migratory birds—cranes, ducks, geese (36), and seagulls (37). The organism is also found in other wild and domestic bird species, as well as in rodents (38). Insects can carry the organism on their exoskeleton (39).

Table. Epidemiologic studies of laboratory-confirmed cases of sporadic campylobacteriosis

| Number | | | | | | | |
|---|---|---|---|---|---|---|---|
| Cases | Controls | Date | Population | Location | Foods associated with illness | Animal contacts | Ref. |
| 52 | 103 | 1989-1990 | Residents of three counties | Norway | Poultry, sausage | Dogs | 27 |
| 218 | 526 | 1982-1983 | HMO patients | Washington State | Undercooked chicken | Animals with diarrhea | 30,34 |
| 29 | 42 | 1990 | Residents of Manchester | England | Bottled milk[a] | | 33 |
| 45 | 45 | 1983-1984 | University students | Georgia | Chicken | Cats | 31 |
| 53 | 106 | 1982-1983 | Rural children | Iowa | Raw milk | | 32 |
| 40 | 80 | 1981 | Residents of Denver, Ft. Collins | Colorado | Untreated water, raw milk, undercooked chicken | Cats | 29 |
| 54 | 54 | 1982 | Residents of Rotterdam | Netherlands | Chicken, pork, barbequed foods | | 28 |
| 10 | 15 | 1982 | Residents of Larimer County | Colorado | Preparing chicken | | 26 |
| 55 | 14 | 1980 | Residents of Göteborg | Sweden | Preparing chicken | Kitten, dog with diarrhea | 25 |

[a]Bottle tops pecked by wild birds.

The intestines of poultry are easily colonized with *C. jejuni*. Day-old chicks can be colonized with as few as 35 organisms (40). Most chickens in commercial operations are colonized by 4 weeks (41,42). Vertical transmission (i.e., from breeder flocks to progeny) has been suggested in one study but is not widely accepted (43). Reservoirs in the poultry environment include beetles (39), unchlorinated drinking water (44), and farm workers (41,42,45). Feeds are an unlikely source of campylobacters since they are dry and campylobacters are sensitive to drying.

*C. jejuni* is a commensal organism of the intestinal tract of cattle (46). Young animals are more often colonized than older animals, and feedlot cattle are more likely than grazing animals to carry campylobacters (47). In one study, colonization of dairy herds was associated with drinking unchlorinated water (48).

Campylobacters are found in natural water sources throughout the year. The presence of campylobacters is not clearly correlated with indicator organisms for fecal contamination (e.g., *E. coli*)(49). In temperate regions, organism recovery rates are highest during the cold season (49,50). Survival in cold water is important in the life cycle of campylobacters. In one study, serotypes found in water were similar to those found in humans (50). When stressed, campylobacters enter a "viable but nonculturable state," characterized by uptake of amino acids and maintenance of an intact outer membrane but inability to grow on selective media; such organisms, however, can be transmitted to animals (51). Additionally, unchlorinated drinking water can introduce campylobacters into the farm environment (44,48).

### *Campylobacter* in the Food Supply

*C. jejuni* is found in many foods of animal origin. Surveys of raw agricultural products support epidemiologic evidence implicating poultry, meat, and raw milk as sources of human infection. Most retail chicken is contaminated with *C. jejuni*; one study reported an isolation rate of 98% for retail chicken meat (52). *C. jejuni* counts often exceed $10^3$ per 100 g. Skin and giblets have particularly high levels of contamination. In one study, 12% of raw milk samples from dairy farms in eastern Tennessee were contaminated with *C. jejuni* (53). Raw milk is presumed to be contaminated by bovine feces; however, direct contamination of milk as a consequence of mastitis also occurs (54). Campylobacters are also found in red meat. In one study, *C. jejuni* was present in 5% of raw ground beef and in 40% of veal specimens (55).

### Control of *Campylobacter* Infection

### On the Farm

Control of *Campylobacter* contamination on the farm may reduce contamination of carcasses, poultry, and red meat products at the retail level (27). Epidemiologic studies indicate that strict hygiene reduces intestinal carriage in food-producing animals (41,42,45). In field studies, poultry flocks that drank chlorinated water had lower intestinal colonization rates than poultry that drank unchlorinated water (42,44). Experimentally, treatment of chicks with commensal bacteria (56) and immunization of older birds (57) reduced *C. jejuni* colonization. Because intestinal colonization with campylobacters readily occurs in poultry flocks, even strict measures may not eliminate intestinal carriage by food-producing animals (39,41).

### At Processing

Slaughter and processing provide opportunities for reducing *C. jejuni* counts on food-animal carcasses. Bacterial counts on carcasses can increase during slaughter and processing steps. In one study, up to a 1,000-fold increase in bacterial counts on carcasses was reported during transportation to slaughter (58). In studies of chickens (59) and turkeys (60) at slaughter, bacterial counts increased by approximately 10- to 100-fold during defeathering and reached the highest level after evisceration. However, bacterial counts on carcasses decline during other slaughter and processing steps. In one study, forced-air chilling of swine carcasses caused a 100-fold reduction in carcass contamination (61). In Texas turkey plants, scalding reduced carcass counts to near or below detectable levels (60). Adding sodium chloride or trisodium phosphate to the chiller water in the presence of an electrical current reduced *C. jejuni* contamination of chiller water by 2 $\log_{10}$ units (62). In a slaughter plant in England, use of chlorinated sprays and maintenance of clean working surfaces resulted in a 10- to 100-fold decrease in carcass contamination (63). In another study, lactic acid spraying of swine carcasses reduced counts by at least 50% to often undetectable levels (64). A radiation dose of 2.5 KGy reduced *C. jejuni* levels on retail poultry by 10 $\log_{10}$ units (65).

### Conclusions

*C. jejuni,* first identified as a human diarrheal pathogen in 1973, is the most frequently diagnosed bacterial cause of human gastroenteritis in the United States. Sequelae including GBS and reactive arthritis are increasingly recognized, adding to the human and economic cost of illness from human campylobacteriosis. The emergence of fluoroquinolone-resistant infections in Europe and the United States, temporally associated with the approval of fluoroquinolone use in veterinary medicine, is also a public health concern. The consumption of undercooked poultry and cross-contamination of other foods with drippings from raw poultry are leading risk factors for human campylobacteriosis. Reinforcing hygienic practices at each link in the food chain—from producer to consumer—is critical in preventing the disease.

Dr. Altekruse is a Public Health Service Epidemiology Fellow with the Food and Drug Administration, Center for Veterinary Medicine. His current research interest is antimicrobial-resistant foodborne pathogens.

### References

1. Kist M. The historical background of *Campylobacter* infection: new aspects. In: Pearson AD, editor. Proceedings of the 3rd International Workshop on *Campylobacter* Infections; Ottawa;1985 Jul 7-10. London: Public Health Laboratory Service;1985. p.23-7.
2. Tauxe RV. Epidemiology of *Campylobacter jejuni* infections in the United States and other industrial nations. In: Nachamkin I, Blaser MJ, Tompkins LS, editors. *Campylobacter jejuni*: current and future trends. Washington: American Society for Microbiology; 1992. p. 9-12.
3. Blaser MJ, Wells JG, Feldman RA, Pollard RA, Allen JR, the Collaborative Diarrheal Disease Study Group. *Campylobacter* enteritis in the United States: a multicenter study. Ann Intern Med 1983;98:360-5.
4. Peterson MC. Clinical aspects of *Campylobacter jejuni* infections in adults. Wes J Med 1994;161:148-52.
5. Sorvillo FJ, Lieb LE, Waterman SH. Incidence of campylobacteriosis among patients with AIDS in Los Angeles County. J Acquir Immune Defic Syndr Hum Retrovirol 1991;4:598-602.
6. Perlman DJ, Ampel NM, Schifman RB, Cohn DL, Patton CM, Aguirre ML, et al. Persistent *Campylobacter jejuni* infections in patients infected with the human immunodeficiency virus (HIV). Ann Intern Med 1988;108:540-6.
7. Allos BM. Association between *Campylobacter* infection and Guillain-Barré syndrome. J Infect Dis 1997;176:S125-8.
8. Peterson MC. Rheumatic manifestations of *Campylobacter jejuni* and *C. fetus* infections in adults. Scand J Rheumatol 1994;23:167-70.
9. Shoenfeld Y, George J, Peter JB. Guillain-Barré as an autoimmune disease. Int Arch Allergy Immunol 1996;109:318-26.
10. Blaser MJ. *Campylobacter* species. In: Principles and practice of infectious diseases. Mandell GL, Douglas RG, Bennett JE, editors. 3rd ed. New York: Churchill Livingstone, 1990;194:1649-58.
11. Murphy GS Jr, Echeverria P, Jackson LR, Arness MK, LeBron C, Pitarangsi C. Ciprofloxacin- and azithromycin-resistant *Campylobacter* causing traveler's diarrhea in U.S. troops deployed to Thailand in 1994. Clin Infect Dis 1996;22:868-9.
12. Piddock LJV. Quinolone resistance and *Campylobacter* spp. Antimicrob Agents Chemother 1995;36:891-8.
13. Jacobs-Reitsma WF, Kan CA, Bolder NM. The induction of quinolone resistance in *Campylobacter* bacteria in broilers by quinolone treatment. In: Campylobacters, helicobacters, and related organisms. Newell DG, Ketley JM, Feldman RA, editors. New York: Plenum Press; 1996. p. 307-11.
14. Smith KE, Besser JM, Leano F, Bender J, Wicklund J, Johnson B, et al. Fluoroquinolone-resistant *Campylobacter* isolated from humans and poultry in Minnesota [abstract]. Program of the 1st International Conference on Emerging Infectious Diseases; Atlanta, Georgia; 1998 Mar 7-10. Atlanta: Centers for Disease Control and Prevention;1998.
15. Blaser MJ, Sazie E, Williams LP Jr. The influence of immunity on raw milk-associated *Campylobacter* infection. JAMA 1987;257:43-6.
16. Black RE, Levine MM, Clements ML, Hughes TP, Blaser MJ. Experimental *Campylobacter jejuni* infection in humans. J Infect Dis 1988;157:472-9.
17. Ketley JM. Pathogenesis of enteric infection by *Campylobacter*. Microbiology 1997;143:5-21.
18. Nachamkin I. *Campylobacter* and *Arcobacter*. In: Manual of clinical microbiology. 6th ed. Washington: ASM Press; 1995. p. 483-91.
19. Humphrey TJ. An appraisal of the efficacy of pre-enrichment for the isolation of *Campylobacter jejuni* from water and food. Journal of Applied Bacteriology 1989;66:119-26.
20. Penner JL, Hennessy JN, Congi RV. Serotyping of *Campylobacter jejuni* and *Campylobacter coli* on the basis of thermostable antigens. Eur J Clin Microbiol Infect Dis 1983;2:378-83.
21. Lior H, Woodward DL, Edgar JA, Laroche LJ, Gill P. Serotyping of *Campylobacter jejuni* by slide agglutination based on heat-labile antigenic factors. J Clin Microbiol 1982;15:761-8.

22. Hilton AC, Mortiboy D, Banks JG, Penn CW. RAPD analysis of environmental, food and clinical isolates of *Campylobacter* spp. FEMS Immunol Med Microbiol 1997;18:119-24.
23. Meinersmann RJ, Helsel LO, Fields PI, Hiett KL. Discrimination of *Campylobacter jejuni* isolates by fla gene sequencing. J Clin Microbiol 1997;35:2810-4.
24. Harrington CS, Thomson-Carter FM, Carter PE. Evidence for recombination in the flagellin locus of *Campylobacter jejuni*: implications for the flagellin gene typing scheme. J Clin Microbiol 1997;35:2386-92.
25. Norkrans G, Svedhem Å. Epidemiologic aspects of *Campylobacter jejuni* enteritis. Journal of Hygiene (Cambridge) 1982;89:163-70.
26. Hopkins RS, Scott AS. Handling raw chicken as a source for sporadic *Campylobacter jejuni* infections [letter]. J Infect Dis 1983;148:770.
27. Kapperud G, Skjerve E, Bean NH, Ostroff SM, Lassen J. Risk factors for sporadic *Campylobacter* infections: results of a case-control study in southeastern Norway. J Clin Microbiol 1992;30:3117-21.
28. Oosterom J, den Uyl CH, Bänffer JRJ, Huisman J. Epidemiologic investigations on *Campylobacter jejuni* in households with primary infection. Journal of Hygiene (Cambridge) 1984;92:325-32.
29. Hopkins RS, Olmsted R, Istre GR. Endemic *Campylobacter jejuni* infection in Colorado: identified risk factors. Am J Public Health 1984;74:249-50.
30. Harris NV, Weiss NS, Nolan CM. The role of poultry and meats in the etiology of *Campylobacter jejuni/coli* enteritis. Am J Public Health 1986;76:407-11.
31. Deming MS, Tauxe RV, Blake PA. *Campylobacter* enteritis at a university from eating chickens and from cats. Am J Epidemiol 1987;126:526-34.
32. Schmid GP, Schaefer RE, Plikaytis BD, Schaefer JR, Bryner JH, Wintermeyer LA, et al. A one-year study of endemic campylobacteriosis in a midwestern city: association with consumption of raw milk. J Infect Dis 1987;156:218-22.
33. Lighton LL, Kaczmarski EB, Jones DM. A study of risk factors for *Campylobacter* infection in spring. Public Health 1991;105:199-203.
34. Saaed AM, Harris NV, DiGiacomo RF. The role of exposure to animals in the etiology of *Campylobacter jejuni/coli* enteritis. Am J Epidemiol 1993;137:108-14.
35. Nielsen EM, Engberg J, Madsen M. Distribution of serotypes of *Campylobacter jejuni* and *C. coli* from Danish patients, poultry, cattle, and swine. FEMS Immunol Med Microbiol 1997;19:47-56.
36. Luetchefeld NA, Blaser MJ, Reller LB, Wang WL. Isolation of *Campylobacter fetus* subsp. *jejuni* from migratory waterfowl. J Clin Microbiol 1980;12:406-8.
37. Glunder G, Neumann U, Braune S. Occurrence of *Campylobacter* spp. in young gulls, duration of *Campylobacter* infection and reinfection by contact. Journal of Veterinary Medicine [Series B] 1992;39:119-22.
38. Cabrita J, Rodrigues J, Braganca F, Morgado C, Pires I, Goncalves AP. Prevalence, biotypes, plasmid profile and antimicrobial resistance of *Campylobacter* isolated from wild and domestic animals from northeast Portugal. Journal of Applied Bacteriology 1992;73:279-85.
39. Jacobs-Reitsma WF, van de Giessen AW, Bolder NM, Mulder RWAW. Epidemiology of *Campylobacter* spp. at two Dutch broiler farms. Epidemiol Infect 1995;114:413-21.
40. Kaino K, Hayashidani H, Kaneko K, Ogawa M. Intestinal colonization of *Campylobacter jejuni* in chickens. Japanese Journal of Veterinary Science1988;50:489-94.
41. Humphrey TJ, Henley A, Lanning DG. The colonization of broiler chickens with *Campylobacter jejuni*; some epidemiologic investigations. Epidemiol Infect 1993;110:601-7.
42. Kapperud G, Skjerve E, Vik L, Hauge K, Lysaker A, Aalmen I, et al. Epidemiological investigation of risk factors for *Campylobacter* colonization in Norwegian broiler flocks. Epidemiol Infect 1993;111:45-55.
43. Pearson AD, Greenwood MH, Feltham RK, Healing TD, Donaldson J, Jones DM, et al. Microbial ecology of *Campylobacter jejuni* in a United Kingdom chicken supply chain: intermittent common source, vertical transmission, and amplification by flock propagation. Appl Environ Microbiol 1996;62:4614-20.
44. Pearson AD, Greenwood M, Healing TD, Rollins D, Shahamat M, Donaldson J, et al. Colonization of broiler chickens by waterborne *Campylobacter jejuni*. Appl Environ Microbiol 1993;59:987-96.
45. Kazwala RR, Collins JD, Hannan J, Crinion RAP, O'Mahony H. Factors responsible for the introduction and spread of *Campylobacter jejuni* infection in commercial poultry production. Vet Rec 1990;126:305-6.
46. Fricker CR, Park RWA. A two year study of the distribution of thermophilic campylobacters in human, environmental and food samples from the Reading area with particular reference to toxin production and heat stable serotype. Journal of Applied Bacteriology 1989;66:477-90.
47. Giacoboni GI, Itoh K, Hirayama K, Takahashi E, Mitsuoka T. Comparison of fecal *Campylobacter* in calves and cattle of different ages and areas in Japan. J Vet Med Sci 1993;55:555-9.
48. Humphrey TJ, Beckett P. *Campylobacter jejuni* in dairy cows and raw milk. Epidemiol Infect 1987;98:263-9.
49. Carter AM, Pacha RE, Clark GW, Williams EA. Seasonal occurrence of *Campylobacter* spp. and their correlation with standard indicator bacteria. Appl Environ Microbiol 1987;53:523-6.
50. Bolton FJ, Coates D, Hutchinson DN, Godfree AF. A study of thermophilic campylobacters in a river system. Journal of Applied Bacteriology 1987;62:167-76.
51. Stern N, Jones D, Wesley I, Rollins D. Colonization of chicks by non-culturable *Campylobacter* spp. Letters in Applied Microbiology 1994;18:333-6.
52. Stern NJ, Line JE. Comparison of three methods for recovery of *Campylobacter* spp. from broiler carcasses. Journal of Food Protection 1992;55:663-6.
53. Rohrbach BW, Draughon FA, Davidson PM, Oliver SP. Prevalence of *Listeria monocytogenes*, *Campylobacter jejuni*, *Yersinia enterocolitica*, and *Salmonella* in bulk tank milk: risk factors and risk of human exposure. Journal of Food Protection 1992;55:93-7.
54. Hudson PJ, Vogt RL, Brondum J, Patton CM. Isolation of *Campylobacter jejuni* from milk during an outbreak of campylobacteriosis. J Infect Dis 1984;150:789.

55. Lammerding AM, Garcia MM, Mann ED, Robinson Y, Dorward WJ, Truscott RB, et al. Prevalence of *Salmonella* and thermophilic *Campylobacter* in fresh pork, beef, veal, and poultry in Canada. Journal of Food Protection 1988;51:47-52.
56. Stern NJ. Mucosal competitive exclusion to diminish colonization of chickens by *Campylobacter jejuni*. Poult Sci 1994;73:402-7.
57. Widders PR, Perry R, Muir WI, Husband AJ, Long KA. Immunization of chickens to reduce intestinal colonization with *Campylobacter jejuni*. Br Poult Sci 1996;37:765-8.
58. Stern NJ, Clavero MRS, Bailey JS, Cox NA, Robach MC. *Campylobacter* spp. in broilers on the farm and after transport. Poult Sci 1995;74:937-41.
59. Izat AL, Gardner FA, Denton JH, Golan FA. Incidence and levels of *Campylobacter jejuni* in broiler processing. Poult Sci 1988;67:1568-72.
60. Acuff GR, Vanderzant C, Hanna MO, Ehlers JG, Golan FA, Gardner FA. Prevalence of *Campylobacter jejuni* in turkey carcasses during further processing of turkey products. Journal of Food Protection 1986;49:712-7.
61. Oosterom J, De Wilde GJA, De Boer E, De Blaauw LH, Karman H. Survival of *Campylobacter jejuni* during poultry processing and pig slaughtering. Journal of Food Protection 1983;46:702-6.
62. Li YB, Walker JT, Slavik MF, Wang H. Electrical treatment of poultry chiller water to destroy *Campylobacter jejuni*. Journal of Food Protection 1995;58:1330-4.
63. Mead GC, Hudson WR, Hinton MH. Effect of changes in processing to improve hygiene control on contamination of poultry carcasses with *Campylobacter*. Epidemiol Infect 1995;115:495-500.
64. Epling LK, Carpenter JA, Blankenship LC. Prevalence of *Campylobacter* spp. and *Salmonella* spp. on pork carcasses and the reduction effected by spraying with lactic acid. Journal of Food Protection 1993;56:536-7,540.
65. Patterson MF. Sensitivity of *Campylobacter* spp. to irradiation in poultry meat. Letters in Applied Microbiology 1995;20:338-40.
